# Leveraging large language models in patient-reported outcome measure development: practical opportunities, cautions, and a human-in-the-loop roadmap

**DOI:** 10.1186/s41687-026-01112-2

**Published:** 2026-06-02

**Authors:** Constance Mara, Tiffany Rybak

**Affiliations:** 1 Behavioral Medicine & Clinical Psychology, Cincinnati Children’s Hospital Medical Center, Cincinnati, USA; 2 Department of Pediatrics, College of Medicine, University of Cincinnati, Cincinnati, USA

## Abstract

**Background:**

Patient-reported outcome (PRO) measure development involves multiple language-intensive stages, including conceptual domain definition, candidate item generation, qualitative refinement, and cognitive interviewing prior to psychometric evaluation. Large language models (LLMs) may offer new opportunities to support these qualitative development activities, although their role within established PRO development frameworks remains incompletely defined.

**Aims:**

This commentary proposes a practical human-in-the-loop roadmap for integrating LLMs into the qualitative phases of PRO development while preserving established standards for content validity and psychometric rigor.

**Approach:**

Drawing on examples from development of the Eating Behavior Measurement (EBM) project and emerging literature from PRO science, survey methodology, and psychological measurement, we outline several bounded use cases for LLMs in PRO measure qualitative development workflows. These include accelerating synthesis of legacy item pools (“domain cartography”), generating candidate items within human-defined constructs, supporting iterative item revision in response to cognitive interview findings, conducting semantic coherence checks for construct alignment, and assisting with developmental or contextual adaptation of candidate items. Across these applications, LLMs function as structured drafting and analytic tools rather than arbiters of validity. We additionally discuss practical risks involving confirmation bias, semantic circularity, transparency, reproducibility, and construct drift, along with strategies for mitigation through human oversight and model triangulation.

**Conclusions:**

LLMs do not replace qualitative inquiry, expert judgment, or empirical psychometric validation. Rather, they may help support more systematic and scalable qualitative development workflows when used within bounded, human-centered measurement frameworks. The central challenge for PRO science is not whether to adopt these tools, but how to integrate them responsibly without compromising the evidentiary standards on which the field depends.

Patient-reported outcome (PRO) measure development involves multiple stages that are fundamentally language-intensive. It begins with concepts expressed in patients’ own words, translating those concepts into domain definitions, drafting candidate items, refining them through expert review and cognitive interviewing, and then quantitatively testing whether the resulting items behave psychometrically as intended. Although multiple methodological traditions guide PRO measure development, contemporary workflows generally involve iterative conceptualization, review of existing measurement approaches, candidate item generation, qualitative refinement, and the subsequent empirical psychometric validation. Cognitive interviewing remains central to this development process because it helps determine whether respondents understand items as intended and whether wording is developmentally appropriate, readable, and translatable [3]. In this commentary, we discuss how large language models (LLMs) may support these broad stages of development prior to quantitative evaluation within established human-centered measurement paradigms. Throughout, we draw on examples from our experience developing the Eating Behavior Measurement (EBM), an ongoing multidomain pediatric measure development effort focused on eating, feeding, and mealtime behaviors among children and their families, to illustrate how these tools may be incorporated into real-world development workflows. We focus specifically on the use of LLMs during qualitative and developmental stages of PRO construction and do not advocate for replacement of conventional psychometric modeling, scoring, or validation frameworks.

LLMs, a class of generative artificial intelligence (AI) systems trained to generate and analyze natural language, are, at base, unusually capable language tools. That does not make them validators of measurement quality, nor does it diminish the need for rigorous human oversight, qualitative inquiry, or empirical psychometric testing. However, it does make them potentially useful across several stages of PRO development that are fundamentally linguistic in nature. Much of contemporary PRO measure development in the early qualitative phases involve tasks such as synthesizing qualitative narratives, defining conceptual domains, drafting and refining candidate items, comparing semantic distinctions across constructs, and iteratively revising wording based on respondent feedback. These activities are not purely psychometric; they are interpretive and language-driven processes requiring repeated translation between lived experience, conceptual meaning, and structured operationalization of intended constructs. As such, LLMs may offer practical utility as structured drafting and evaluation tools during the qualitative development phase, even if they cannot determine whether a measure is ultimately valid.

Recent PRO commentaries have begun to make that case explicitly, arguing that generative AI may help the field rethink how measures are designed, administered, and interpreted, while still requiring rigorous human oversight and validation [1, 11]. Parallel work in survey methodology similarly argues that LLMs may help uncover linguistic ambiguities, conceptual overlap, and underexplored themes earlier in instrument development rather than after large-scale field testing [5]. However, whereas prior discussions of this topic have emphasized conceptual transformation or future-facing possibilities, our focus is more pragmatic: how LLMs may be operationally integrated into contemporary qualitative development workflows in bounded, human-in-the-loop ways that support established PRO measure development frameworks rather than replace them.

A practical place to start is the earliest phase of measure development: understanding the existing measurement landscape. In many domains, investigators begin by reviewing dozens of legacy measures, extracting hundreds or thousands of items, and determining what is already measured well, what is measured inconsistently, and what is not measured at all. In our own EBM work, we reviewed more than 80 existing measures and over 1,400 items, not because all of those items would be retained, but because they served as a map of the field. They demonstrated which caregiver, child, and contextual feeding, eating, and mealtime constructs appeared repeatedly, which were fragmented across scales, and which were underrepresented or poorly operationalized. That kind of “domain cartography” is exactly the sort of task LLMs may help accelerate. They can summarize large item sets, propose candidate construct clusters, flag semantic overlap, and generate draft operational definitions for human review. Using LLMs in this manner does not eliminate the need for investigator judgment or theoretical foundation, but it allows measure development teams to spend less time sorting and more time critically evaluating. This kind of LLM-assisted survey development has been proposed explicitly in recent health-methods work [5].

A second and more obvious use case is candidate item generation. The automatic item generation literature predates current LLMs, but recent reviews show that LLMs have rapidly expanded what is feasible in this space. A 2025 review of LLM-based automatic item generation identified 60 relevant studies and concluded that LLMs are increasingly being used to generate and refine candidate items, although, again, human review and empirical evaluation remain essential [10]. In other words, the important contribution is not simply that “AI can write items.” It is that generative AI can expand the candidate item space. In our EBM work, that is how we used it: as a structured brainstorming tool to draft items in places where existing measures suggested conceptual gaps or where legacy item wording seemed poorly aligned with the construct we wanted to capture. For example, many existing measures included highly specific questions about who fed a child during particular meals or on specific days of the week. Through expert review and iterative prompting, we used LLM-assisted item generation to help draft broader candidate items focused instead on the underlying construct of interest, such as the extent to which children typically eat alone, eat with caregivers present, or participate in consistent mealtime routines. Similarly, rather than focusing narrowly on counts of specific foods consumed, we explored candidate items assessing whether caregivers perceived nutritious foods as available, offered, and incorporated into routine feeding practices. In this context, LLMs functioned less as autonomous item writers and more as structured brainstorming tools that helped expand possible ways of operationalizing complex feeding constructs across developmental stages. Used this way, LLMs can generate alternative phrasings, vary examples, adjust reading level, and expose whether a construct definition is sufficiently precise to support item writing in the first place.

A third use case is item revision. Questionnaire development is full of small but consequential wording decisions: whether “healthy” should become “nutritious,” whether “food or drink” is too broad, whether examples are needed, whether “upset” is too vague relative to “frustrated,” “anxious,” or “overstimulated.” These are the kinds of issues that traditionally surface through cognitive interviewing. Modern PRO measure development processes treat cognitive interviewing as essential because it reveals how respondents understand item language, retrieve relevant experiences, form judgments, and map those judgments onto responses [4]. LLMs may be useful here as bounded drafting partners. They can rapidly produce alternative phrasings in response to interview findings, suggest clearer contextual examples, or generate developmentally tailored variants for expert and respondent review. For example, in our EBM work, when refining items assessing caregiver provision of healthy food options, we found that candidate phrasings drawn from existing literature and expert brainstorming often felt overly evaluative or judgment-laden when presented to families. We used LLMs to generate alternative wording and examples that preserved the intended construct while softening tone and reducing perceived stigma. One suggestion involved replacing the term healthy with nutritious, which we subsequently evaluated alongside other alternatives through expert review and family feedback. Families consistently viewed nutritious as less judgmental and less stigmatizing, ultimately informing our final item wording decisions. The logic is similar to what Kuru [5] describes for broader health survey design: LLMs can support, but not replace, the iterative refinement process.

A fourth use case, and one we found especially useful, is semantic coherence checks for construct alignment. In our own applied work, we not only used LLMs to help generate items; we also fed item sets back to the model without their construct labels and asked what operational definition the items appeared to represent (i.e., “reverse-engineered” the operational definition). Framed carefully, this is not “AI validation.” It is closer to a semantic coherence check. If a set of items intended to reflect emotional feeding is inferred by LLMs to represent instrumental feeding, or if two supposedly distinct subdomains elicit nearly identical inferred definitions, that is a useful signal that wording may not clearly separate the constructs. When feasible, semantic coherence checks may be strengthened by conducting secondary construct-inference tasks using different model architectures than those used during earlier item-generation stages.

Emerging psychometric research points in a similar direction. Milano et al. [7] showed that LLM embeddings can recover a-priori factorial structure from item semantics, and Milano et al. [8] found that LLM-based construct mapping may complement human content-validity judgments, although performance depends on item type and human expertise remains crucial. These studies are not PRO-specific, but they are highly relevant to the idea that semantic structure can provide an upstream check on conceptual alignment before response data are collected. Importantly, semantic similarity between items should not be conflated with psychometric equivalence or construct validity. LLMs may identify linguistic overlap, conceptual proximity, or semantic clustering within item content, but these patterns do not establish dimensionality, invariance, reliability, or other psychometric properties. At most, such evaluations from LLMs may function as upstream qualitative aids that help investigators identify potential ambiguities, redundancies, or construct overlap prior to empirical testing. The ultimate evaluation of measurement performance remains a quantitative and empirical process.

A fifth use case is adaptation and tailoring. PRO measure development teams often need to modify instruments for developmental stage, disease context, care setting, respondent type, literacy level, or cultural context. LLMs may help teams draft candidate adaptations more quickly than starting from scratch each time. For example, an item assessing children’s recognition of hunger and fullness cues may require substantially different wording depending on developmental stage. Items appropriate for caregivers of infants or toddlers may need to focus on observable behavioral indicators such as turning away from food, refusing bites, or pushing food away, whereas items for older children may refer more directly to children verbally expressing hunger, fullness, or food preferences. Similarly, feeding situations relevant to adolescents may differ substantially from those relevant to preschool-aged children. In these contexts, LLMs may help generate developmentally tailored candidate wording and contextual examples, but human review remains essential to ensure that adaptations preserve the intended construct rather than subtly shifting its meaning, tone, or difficulty. Notably, any LLM-generated adaptation should be treated as a draft requiring the same qualitative review, translatability review, and empirical testing as any other modified item set [9, 12]. Kuru [5] also explicitly argues that generative AI can support survey adaptation and early-stage refinement, but only if these processes remain transparent and methodologically grounded.

Beyond current applications in development workflows, some authors have proposed more speculative future roles for LLMs in individualized PROM administration and adaptive assessment architectures [1, 11]. While conceptually intriguing, these approaches remain early-stage and raise substantial unresolved questions regarding transparency, calibration, standardization, and psychometric rigor. As such, our focus here remains on bounded applications of LLMs within existing development paradigms in the qualitative phases of PRO development rather than replacement of conventional measurement frameworks.

The risks of using LLMs in early qualitative phases of PRO development, however, are not incidental. LLMs can hallucinate constructs, collapse meaningful distinctions between related domains, reproduce biases embedded in their training data, and generate wording that sounds polished but is conceptually weak. In our own experience, model-generated suggestions frequently required substantial vetting, as outputs were at times overly broad, semantically imprecise, or subtly misaligned with the intended construct despite appearing superficially plausible. In some cases, model-generated revisions broadened constructs in ways that reduced conceptual specificity or developmental appropriateness despite superficially improving readability or tone, requiring substantial iterative human review and refinement. For example, some model-generated revisions broadened caregiver feeding constructs into general parenting behaviors, reducing conceptual specificity despite improved readability.

Boyer et al. [1] explicitly identify validation, equity, and trust as central challenges for generative AI in PROs. Broader healthcare AI work similarly emphasizes that generative models require careful validation before clinical or research deployment [2]. Additional practical risks include confirmation bias if investigators overinterpret model-generated summaries or draft operational definitions, particularly when early semantic clustering is mistaken for definitive conceptual structure rather than a provisional aid prior to empirical psychometric validation stages of PRO development. Similar concerns arise when LLMs are used for semantic coherence checks, as repeated use of the same model or conversational context may introduce circularity if prior prompts influence later outputs. To mitigate these risks, LLM-generated outputs should be treated as provisional drafts subject to independent theoretical review, expert adjudication, and iterative refinement rather than accepted as authoritative representations of construct boundaries. Of course, following this qualitative process with standard quantitative psychometric phases further refines construct validity. When feasible, investigators may further reduce circularity by using fresh sessions, blinded prompting, or alternative model architectures when conducting secondary semantic review tasks.

Indeed, another practical safeguard is model triangulation. If LLMs are used for semantic clustering, reverse-engineering construct operationalization from item sets, or gap identification, investigators could compare outputs across more than one model architecture (e.g., GPT-class models, CoPilot, Claude, Gemini) to determine whether construct interpretations converge. Divergent inferences may signal unstable semantic structure or ambiguous item wording requiring human review. Such triangulation does not establish validity, but it may reduce overreliance on any single model’s idiosyncrasies.

Beyond construct accuracy, PRO development introduces a distinct and field-specific concern: data privacy. Instrument development frequently involves qualitative datasets containing verbatim patient or caregiver narratives. Feeding cognitive interview transcripts or open-ended responses into commercial LLM platforms may raise questions about data retention, model training reuse, institutional policy alignment, and IRB compliance. Even when de-identified, narrative data may carry contextual identifiers. Investigators considering generative AI-assisted qualitative synthesis should therefore attend to data governance policies, use secure or enterprise deployments when available, and document clearly how sensitive material is handled. The promise of LLMs in PRO science does not eliminate longstanding ethical obligations to protect respondent confidentiality.

Additional concerns involve reproducibility, transparency, and governance of LLM-assisted workflows themselves. Unlike conventional psychometric procedures, many contemporary LLM systems are proprietary, versioned, and only partially transparent to investigators. As a result, identical prompts may yield different outputs across time, platforms, model versions, or deployment environments. Related concerns have also emerged in the broader educational and psychological assessment literature, particularly regarding distinctions between proprietary and open-source LLM systems and their implications for transparency, auditability, and reproducibility within assessment workflows [6]. These issues are especially important in measurement contexts, where standardization, interpretability, and reproducibility remain central methodological priorities. Accordingly, investigators incorporating LLMs into qualitative development workflows should document model architectures, prompting strategies, and major iterative decision points whenever feasible in order to preserve transparency and auditability of construct evolution over time.

Taken together, these risks suggest not retreat, but structure. The question is not whether LLMs should be used in PRO development, but how they can be integrated responsibly within established validity frameworks. Further, researchers should document when and how LLMs are used in item development to preserve transparency and auditability of construct evolution.

With these cautions and limitations in mind, we propose a human-in-the-loop roadmap to leverage LLMs in the early qualitative phases of PRO development (see Fig. 1; Table 1). Start with the usual conceptual work: literature review, extant measure review, expert input, and where possible concept elicitation. Use LLMs to help summarize legacy item pools, identify semantic clusters, and draft candidate operational definitions. Then use the model to generate candidate items or alternate phrasings only after the construct has been clearly defined by humans. Subject those items to the usual filters: expert review, readability and translatability review, and cognitive interviewing with the target population. Use the model again, if helpful, to generate revised alternatives in response to interview findings or to perform semantic-coherence checks on whether item sets still communicate the intended construct. Only then move to the quantitative phase of psychometric testing, where the core questions remain empirical: dimensionality, local dependence, differential item functioning, information, responsiveness, and interpretability. This sequence preserves what contemporary PRO measure development frameworks already recognize: validity is built through cumulative evidence, not through elegant wording alone [3, 4].

**Fig. 1:**
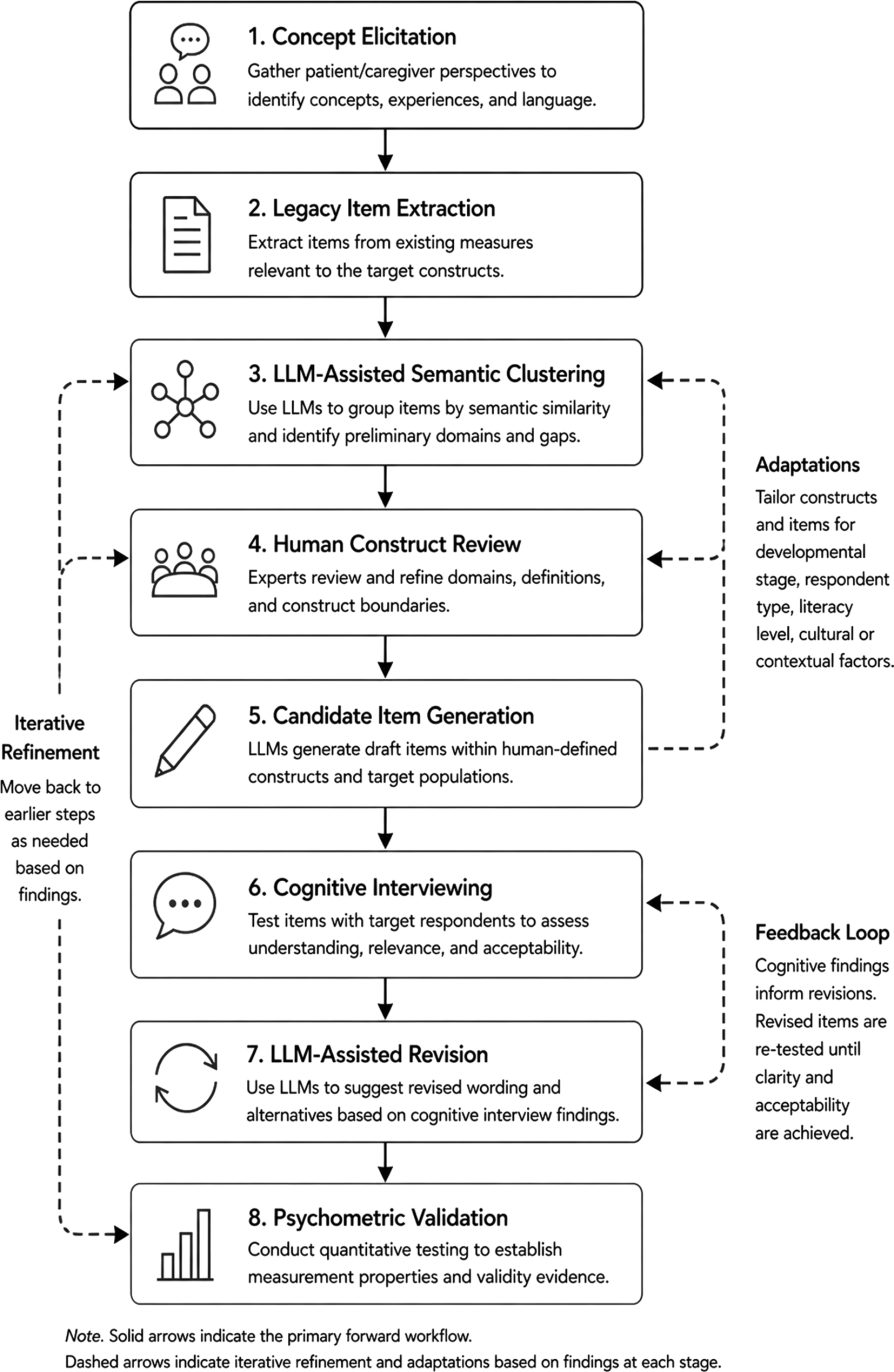
Illustrative human-in-the-loop workflow for integrating LLMs into qualitative phases of PRO measure development

**Table 1:** Practical human-in-the-loop workflow for integrating LLMs into qualitative phase of PRO development

Stage	Human Goal	Potential LLM Contribution	Example Workflow Tasks	Human Oversight Required
1. Conceptual Grounding	Establish theoretically and clinically meaningful constructs	Minimal or none. LLMs may assist with literature summarization but do not define constructs.	Summarize broad topic areas from literature reviews; organize existing conceptual definitions for team review.	Literature review, expert input, concept elicitation, theoretical construct definition.
2. Domain Cartography	Map the existing measurement landscape and identify conceptual gaps	Summarize legacy item pools, identify semantic overlap, propose preliminary construct clusters, draft candidate operational definitions.	Group similar items across measures; identify underrepresented domains; highlight potentially redundant constructs.	Review and refine construct groupings; determine conceptual boundaries and omissions; reject inappropriate clustering.
3. Bounded Candidate Item Generation	Expand candidate item pools within predefined human-defined constructs	Generate draft items, alternative phrasings, contextual examples, and reading-level variants constrained to specified constructs and developmental stages.	Draft caregiver-report items for children of different ages; generate non-stigmatizing alternatives; vary contextual examples while preserving construct focus.	Screen for conceptual alignment, developmental appropriateness, redundancy, tone, bias, and unintended construct drift.
4. Iterative Qualitative Refinement	Improve clarity, interpretability, and acceptability of candidate items	Generate revised wording in response to cognitive interview findings; suggest clearer contextual examples or simplified language.	Revise items perceived as judgmental, vague, overly broad, or developmentally inappropriate; generate multiple alternative phrasings for review.	Cognitive interviewing, readability review, translatability review, expert adjudication, respondent feedback.
5. Semantic Coherence Checks	Examine whether item sets appear to communicate intended constructs	Infer candidate operational definitions from unlabeled item pools; identify semantic overlap across subdomains.	Compare whether item sets intended to represent distinct constructs elicit distinguishable inferred definitions; identify potentially ambiguous or overlapping wording.	Compare inferred definitions against intended constructs; revise items to improve conceptual differentiation. *Semantic analyses do not replace psychometric validation.
6. Adaptation and Tailoring	Adapt candidate items for developmental stage, respondent type, literacy level, or context	Generate developmentally tailored wording and contextually relevant examples while preserving predefined construct targets.	Generate toddler-, child-, adolescent-, or caregiver-specific wording variants; simplify reading level; adapt examples for care settings or populations.	Evaluate whether adaptations preserve intended construct meaning, comparability, tone, and developmental specificity.
7. Model Triangulation and Documentation	Reduce overreliance on a single model architecture and improve transparency	Replicate semantic analyses across multiple LLM systems; document prompting strategies and major iterative decisions.	Compare outputs across GPT-class models, Claude, Gemini, or Copilot; evaluate convergence and divergence across model outputs.	Treat divergence as a signal requiring further review; document model versions, prompting approaches, and iterative revisions to preserve auditability.
8. Quantitative Psychometric Validation	Establish measurement properties and validity evidence	None. LLMs do not replace empirical psychometric testing.	Conduct field testing and quantitative analyses.	Evaluate dimensionality, reliability, local dependence, differential item functioning, responsiveness, interpretability, and validity evidence empirically.

In our own experience applying these principles during the development of the EBM project, this framing has proven useful. We did not use AI to decide whether a construct existed or to declare that an item was valid. We used LLMs in bounded ways: to help think through domain structure, to identify and draft items for undermeasured areas, to propose revisions in response to feedback from cognitive interviews with families, and to reverse-engineer operational definitions to assess whether item sets semantically pointed back to the constructs we intended. At no stage did LLMs provide evidence of validity; rather, they supported earlier qualitative processes that preceded and informed empirical validation. The real validity work still came from the slower, well-established methods of reviewing the measurement landscape and theoretical foundations, convening experts, conducting cognitive interviews, operationally defining constructs, and iteratively revising wording in response to actual caregivers. In that sense, the lesson from our experience is not that generative AI replaces PRO development. It is that LLMs can make rigorous PRO development more systematic, more explicit, and more ambitious.

That, in our view, is the most promising way to think about LLMs in measure development today. Not as autonomous instrument designers or psychometric authorities, and not as replacements for qualitative inquiry or psychometric validation. Rather, as powerful language tools that may help us search wider, draft faster, compare more alternatives, and interrogate semantic structure more deliberately than we could before. For a field built on translating lived experience into valid measurement, that is a meaningful opportunity. The challenge is to use it in ways that expand our methodological reach without compromising the evidentiary standards on which PRO science depends.

## Data Availability

No datasets were generated or analysed during the current study.
